# Efficiency Evaluation of Sampling Density for Indoor Building LiDAR Point-Cloud Segmentation

**DOI:** 10.3390/s25206398

**Published:** 2025-10-16

**Authors:** Yiquan Zou, Wenxuan Chen, Tianxiang Liang, Biao Xiong

**Affiliations:** 1 School of Civil Engineering, Architecture and the Environment, Hubei University of Technology, 28 Nanli Road, Wuhan 430068, China; zouyq@mail.hbut.edu.cn (Y.Z.);; 2 School of Computer Science and Artificial Intelligence, Wuhan University of Technology, 122 Luoshi Road, Wuhan 430070, China

**Keywords:** LiDAR, indoor building mapping, point cloud semantic segmentation, sampling density, uniform down-sampling, optimal density selection, Scan-to-BIM

## Abstract

Prior studies on indoor LiDAR point-cloud semantic segmentation consistently report that sampling density strongly affects segmentation accuracy as well as runtime and memory, establishing an accuracy–efficiency trade-off. Nevertheless, in practice, the density is often chosen heuristically and reported under heterogeneous protocols, which limits quantitative guidance. We present a unified evaluation framework that treats density as the sole independent variable. To control architectural variability, three representative backbones—PointNet, PointNet++, and DGCNN—are each augmented with an identical Point Transformer module, yielding PointNet-Trans, PointNet++-Trans, and DGCNN-Trans trained and tested under one standardized protocol. The framework couples isotropic voxel-guided uniform down-sampling with a decision rule integrating three signals: (i) accuracy sufficiency, (ii) the onset of diminishing efficiency, and (iii) the knee of the accuracy–density curve. Experiments on scan-derived indoor point clouds (with BIM-derived counterparts for contrast) quantify the accuracy–runtime trade-off and identify an engineering-feasible operating band of 1600–2900 points/m^2^, with a robust setting near 2400 points/m^2^. Planar components saturate at moderate densities, whereas beams are more sensitive to down-sampling. By isolating density effects and enforcing one protocol, the study provides reproducible, model-agnostic guidance for scan planning and compute budgeting in indoor mapping and Scan-to-BIM workflows.

## 1. Introduction

Indoor LiDAR point clouds are routinely used for BIM-aligned as-built modeling, quality inspection, and progress tracking; consequently, accurate and efficient semantic segmentation of structural elements is required.

In recent years, point clouds acquired via terrestrial laser scanning have been widely adopted in the architecture, engineering, and construction domain to support digital modeling, progress tracking, and quality verification of buildings [1]. With deep-learning-based semantic segmentation, structural elements such as walls, floors, beams, and columns can be automatically identified and aligned with BIM data, substantially improving information acquisition and inspection efficiency. However, segmentation performance is highly sensitive to sampling density: inadequate density leads to loss of geometric detail and inter-class confusion, while excessive density inflates data volume and computational cost; in practice, density is often chosen heuristically and lacks quantitative justification [2,3]. Hence, striking a balance between accuracy and processing efficiency—by suppressing redundant points without undermining recognition quality—remains a pressing challenge. Prior efforts have begun to address this trade-off: modestly reducing point-cloud resolution can markedly improve throughput with only limited accuracy degradation [4]. Other studies leverage data augmentation (e.g., diffusion-based synthetic indoor point clouds) to enhance training and segmentation robustness [5]. Even so, building scenarios still lack systematic approaches to determine the optimal sampling density with joint consideration of both accuracy and efficiency.

Within learning-based semantic segmentation for buildings, prior work has explored three complementary avenues.

Existing studies generally proceed along three directions. From the model perspective, feature representation is enhanced to raise the performance ceiling, but input density is usually treated as a fixed condition.

From the sampling perspective, voxel-, grid-, or Poisson-based strategies are used to control data scale and speed, yet thresholds remain mostly empirical and evaluations often focus only on a single accuracy curve. This practice makes it difficult to incorporate runtime and memory constraints.

From the data perspective, robustness is improved through synthesis or augmentation, but a unified, protocol-consistent quantitative comparison of the density–accuracy–efficiency relationship is lacking. These advances lay the groundwork for scene understanding, but gaps remain in computable density decision-making and cross-model comparability.

To address these issues, this study explicitly isolates sampling density as the sole independent variable. Under a unified protocol for data preprocessing, training, and evaluation, we build an assessment chain with dual perspectives of task performance and computational efficiency: the former measures segmentation quality, and the latter evaluates benefit per unit processing time.

**Figure 1:**
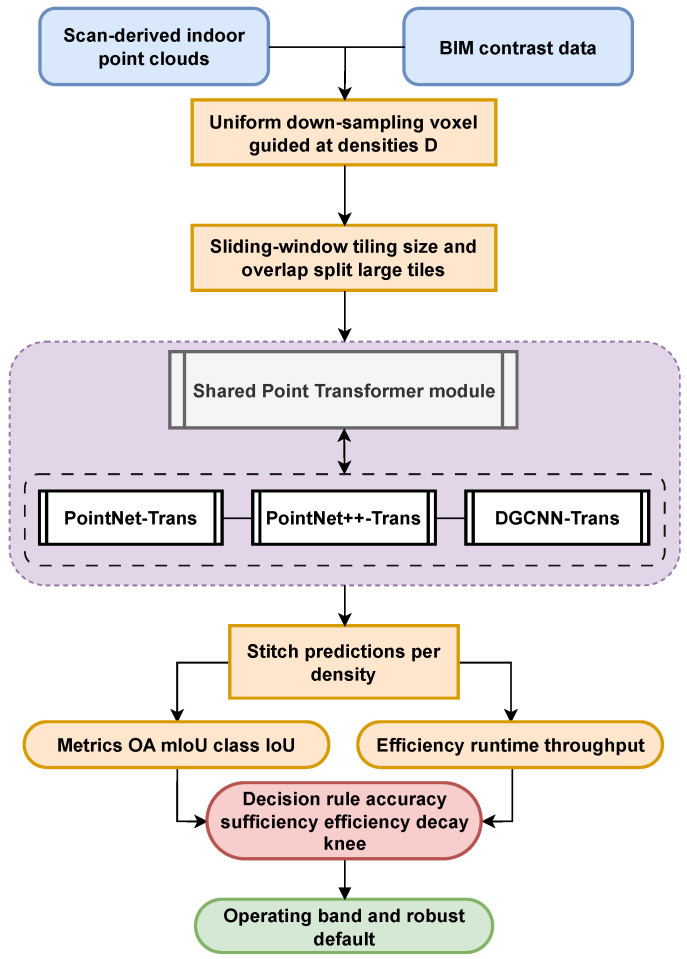
Unified pipeline and density-selection rule for indoor LiDAR.

We further propose a procedural selection criterion consisting of three conditions—whether accuracy meets the required standard, whether efficiency shows decay, and whether the response curve exhibits a structural knee—thereby converting heuristic settings into computable and auditable engineering decisions. The proposed design is independent of any specific backbone, providing a model-agnostic and transferable method for density selection that offers directly actionable guidance for scan planning and inference deployment. Compared with prior density–accuracy analyses, our contribution is a unified, backbone-controlled evaluation together with an explicit, auditable rule for selecting an engineering-feasible operating band. The design is model-agnostic and transferable, while the present scope (single site and primary scanner) is acknowledged and discussed later in the paper. A block diagram of the unified evaluation pipeline and the three-condition decision rule is provided in Figure 1 for quick reference. Our goal is engineering guidance rather than a new backbone or theory; the novelty is to treat density as the sole independent variable under one protocol and to convert the trade-off into a script-implemented three-condition selector that yields a cross-backbone operating band for indoor Scan-to-BIM.

This study (i) establishes a unified evaluation protocol that controls architectural variability across representative backbones; (ii) formulates a three-condition, auditable decision rule that operationalizes density selection; and (iii) quantifies the accuracy–runtime trade-off to indicate an engineering-feasible density band and a robust default. Advantages include reproducibility and model-agnostic applicability to planning and budgeting. Limitations include the single-site, single-scanner validation and the absence of newer SOTA architectures as baselines; these aspects are analyzed and discussed in the following  sections.

The remainder of the paper is organized as follows. We first review relevant theories and prior studies; next, we detail the proposed methodology—including point-cloud processing, training, and the evaluation protocol for optimal density—then we present the experimental validation. We subsequently discuss the method’s effectiveness, limitations, and avenues for future work, and finally we conclude the study.

## 2. Related Work

### 2.1. Applications of Point Clouds in Building Scenarios

In recent years, applications of deep learning to building-scene point clouds have advanced rapidly. Survey studies indicate that point clouds show notable potential for 3D modeling, quality inspection, and progress tracking, while also facing challenges in model performance and data processing [6,7]. Some studies have proposed semantic segmentation networks specifically designed for indoor building scenes. Combined with transfer learning or domain adaptation strategies, these methods maintain stable performance across different projects and help produce accurate as-built BIM models [8]. Other research employs diffusion models to synthesize indoor point clouds, thereby expanding the training set, significantly reducing annotation costs, and improving segmentation accuracy [5]. In addition, leveraging the neighborhood context of BIM components for classification tasks has been shown to improve accuracy and robustness in complex models [9].

In the construction domain, point clouds have been applied to automatic progress monitoring and quality inspection. For example, one study built an as-built BIM reconstruction dataset from real construction-site point clouds, providing a benchmark for point-cloud recognition and BIM alignment [10]. Other studies tackled the problem via automated reconstruction and wall-topology modeling to reduce manual intervention and address missing data [11,12]. Moreover, improvements in efficiency and accuracy have been demonstrated for large-scale construction scenarios through advanced algorithmic frameworks and better data preprocessing [13,14]. Additionally, point-cloud segmentation methods have been developed for quality inspection of prefabricated components, enabling automatic verification of dimensional accuracy [15]. Although the use of point clouds in construction continues to expand, further improvements in data processing efficiency and semantic accuracy are needed under practical constraints.

Due to the difficulty and high cost of acquiring and manually labeling construction-site point clouds, recent research has explored training with virtual point clouds. An automated synthetic point-cloud generation method has successfully trained models and verified the feasibility and stability of this approach [16]. Indoor point clouds and semantic labels generated from BIM can enrich training data and improve generalization to real scenes [17]. Furthermore, point cloud–BIM integration fuses geometric and semantic features for high-precision segmentation and automated progress tracking [18,19].

In summary, recent applications demonstrate tangible benefits across modeling, inspection, and progress tracking, yet density choices remain largely heuristic under practical constraints. This motivates our study to elevate sampling density to a primary, auditable decision variable under a unified protocol and to offer actionable guidance for engineering deployment.

### 2.2. Sampling Density and Efficiency

High-density point clouds contain richer detail but cause surges in storage and computation demand; thus, a trade-off between sampling density and processing efficiency is needed.

For building and BIM processing scenarios, studies have proposed efficient down-sampling and data simplification approaches: improved voxel downsampling markedly reduces point count while preserving geometric features as much as possible [20]; selective sampling based on edges and semantic keypoints retains critical information to the greatest extent [21]; dynamic voxel filtering achieves fast processing under high simplification ratios [22]; and octree partitioning together with a two-level spatial strategy improves out-of-core and block-processing efficiency for very large scenes [23,24].

These methods strike a balance between geometric fidelity and compression rate, substantially enhancing the tractability of large-scale point clouds.

Further, some efforts focus specifically on the efficiency of the sampling process itself. For example, a dynamic Farthest Point Sampling (FPS) method introduces an adaptive multi-level grid to greatly accelerate the classical FPS algorithm. On large-scale point clouds with millions of points, this dynamic FPS (DFPS) preserves global geometry even at very low sampling rates and increases sampling speed by several orders of magnitude [25]. Real-time quality assessment in mobile mapping uses density and completeness as indicators to guide scan trajectories and parameter settings. This ensures sufficient point density in critical regions within limited time and improves the balance between efficiency and accuracy [26]. Feature-preserving adaptive simplification extracts building edges from multi-view imagery to guide reduction, maintaining high geometric accuracy and visual fidelity even at high simplification ratios [27]. Geometry compression based on voxel clustering and deep entropy coding reduces transmission costs while preserving spatial structure, enabling cloud transmission and real-time rendering [28].

From a general-strategy perspective, simplification via geometric feature extraction and clustering maintains the primary shape across different compression ratios: constructing Delaunay neighborhoods and preferentially retaining edge/ridge points helps avoid holes and preserves structural integrity [29]. Using four boundary features, including normal and curvature, to discriminate and then preferentially retain contours and sharp edges maintains boundary details after compression [30]. An improved fuzzy C-means-based partitioning simplification balances local structure preservation and compression during downsampling [31].

In addition, learning-based sampling enables on-demand point selection: curvature- and probabilistic-membership-based partitioning promotes balanced sampling across sparse and dense regions [32]; self-attention-based learned sampling adapts point selection to varying input scales while maintaining reconstruction accuracy at high compression ratios [33]; and combining attention-based edge sampling with FPS preserves contour keypoints more precisely, reducing about 85% of points while achieving improved segmentation accuracy and more efficient training [34]. Overall, whether rule-based or learning-based, a well-designed downsampling strategy can substantially reduce the number of points while preserving key geometric information, thus providing a lighter input for subsequent 3D reconstruction, object classification, and semantic segmentation tasks.

Overall, the above strategies highlight the persistent tension between geometric fidelity and efficiency, but the choice of density thresholds remains ad hoc. Our framework elevates density to a controlled, model-agnostic variable and formalizes a three-condition rule that links accuracy sufficiency to efficiency turning points within one evaluation protocol.

### 2.3. Impact of Density on Segmentation

Point cloud density directly affects the accuracy and efficiency of semantic segmentation, and it can be treated as a controllable variable. For example, adopting a density-ordered sampling strategy has been shown to improve segmentation accuracy [35]. Overall, empirical results indicate a “density threshold effect”: as long as the primary geometry is preserved, moderate density reduction has limited impact on accuracy, but performance drops rapidly once the threshold is crossed [36,37]. This implies that, in engineering practice, one should identify and follow a reasonable density band to significantly reduce computational overhead under an acceptable accuracy loss.

To handle uneven density distributions in large scenes, researchers have explored density-adaptive methods at both the preprocessing and model levels. For example, a density-based adaptive grid downsampling approach dynamically adjusts the voxel size according to local point density, maintaining efficiency while balancing class distribution and achieving high mIoU on public datasets [3].

Additionally, parallel multi-resolution networks combined with density-aware feature weighting can mitigate instabilities caused by abrupt local density changes, achieving better accuracy with fewer parameters [38]. Moreover, multi-scale voxel–point fusion networks use larger voxels to filter out redundant points while retaining raw point features at fine detail, yielding greater robustness for large-scene segmentation [39]. An adaptive spatial-structure graph Transformer optimizes dynamic graph connectivity to alleviate interference from point clustering and occlusions in high-density regions, further improving overall completeness and accuracy [40]. Meanwhile, adopting advanced segmentation architectures—such as Transformer-based models [41] and local–global feature fusion frameworks [42]—further enhances the efficiency of large-scale point-cloud semantic segmentation.

In sum, appropriate downsampling strategies combined with density-adaptive network architectures can substantially reduce data volume while maintaining segmentation accuracy on primary targets, thereby enabling the search for an “optimal sampling density” that balances performance and cost within task requirements.

Taken together, prior observations suggest the existence of a practical density band and a knee in the accuracy–density curve; however, consistent cross-model criteria are rarely specified. We operationalize this by jointly quantifying accuracy and marginal efficiency under a unified protocol and by selecting an engineering-feasible operating band via a procedural, auditable rule. Instead of designing new efficient backbones (e.g., RandLA-Net) or attention-based architectures (e.g., Point Transformer), we contribute a protocol-level, model-agnostic selector that standardizes density choice for deployment.

## 3. Materials and Methods

### 3.1. Overall Research Framework

This section outlines the overall research framework, which is centered on the core issue of “sampling density–accuracy–efficiency.” We build an end-to-end pipeline that spans from data generation to determining the optimal sampling density. In summary, both “virtual” and “real” point clouds are derived in parallel from BIM models and a unified semantic segmentation model (three representative backbones plus a Transformer) is applied under a consistent training protocol. We then perform standardized density evaluation and efficiency measurement to identify the optimal density along with a recommended range for engineering deployment and computational budgeting.

On the data side, we utilize both virtual and real point clouds as complementary sources. BIM models are first exported to FBX (via Autodesk Revit 2024), converted into high-density virtual point clouds, and tiled to form controllable, large-scale training samples. In parallel, real on-site point clouds are captured, registered, denoised, and manually annotated to serve as an independent test set and a basis for comparison. This dual data-source design balances controllability and real-world transferability, ensuring that the conclusions drawn are practically generalizable.

On the model and training side, we select PointNet, PointNet++, and DGCNN as representative backbones and uniformly integrate a Point Transformer to form a consistent “three-backbone + Transformer” segmentation paradigm. The training protocol is fully aligned across all models in terms of data augmentation, input point count normalization, optimization and learning rate schedule, EMA usage, and early stopping. This alignment ensures that sampling density is the only independent variable under comparison, rather than architectural differences.

In implementation, the three models are interchangeable via configuration. The input pipeline uses a custom collate_fn to pad or crop each sample to a fixed number of points *N*, ensuring a consistent point count per batch and balanced GPU memory usage. During inference, floor-level point clouds are processed using a tiling approach with overlapping sliding windows, and a voting-based fusion is applied to overlapping regions to restore full-floor semantic outputs. This approach provides consistent results for density evaluation and error analysis [43,44].

On the evaluation side, we establish a unified density assessment protocol. We generate a series of uniformly downsampled point-cloud levels by combining a voxel grid method with a binary search procedure. For each downsampling level, sliding-window inference and reconstruction are performed; we record composite accuracy metrics, processing time, and memory usage, and compute the marginal efficiency. Using three types of evidence—accuracy sufficiency, efficiency decay, and curve knee point—we then automatically identify an optimal sampling density for each model. Finally, we aggregate the results from the three models to obtain a recommended density interval for indoor building scenes.

Compared to random sampling, uniform downsampling distributes points more evenly in 3D space and is highly reproducible. It reduces the point count while preserving the overall geometric skeleton and primary structural features, thereby ensuring comparability of results across different densities.

**Figure 2:**
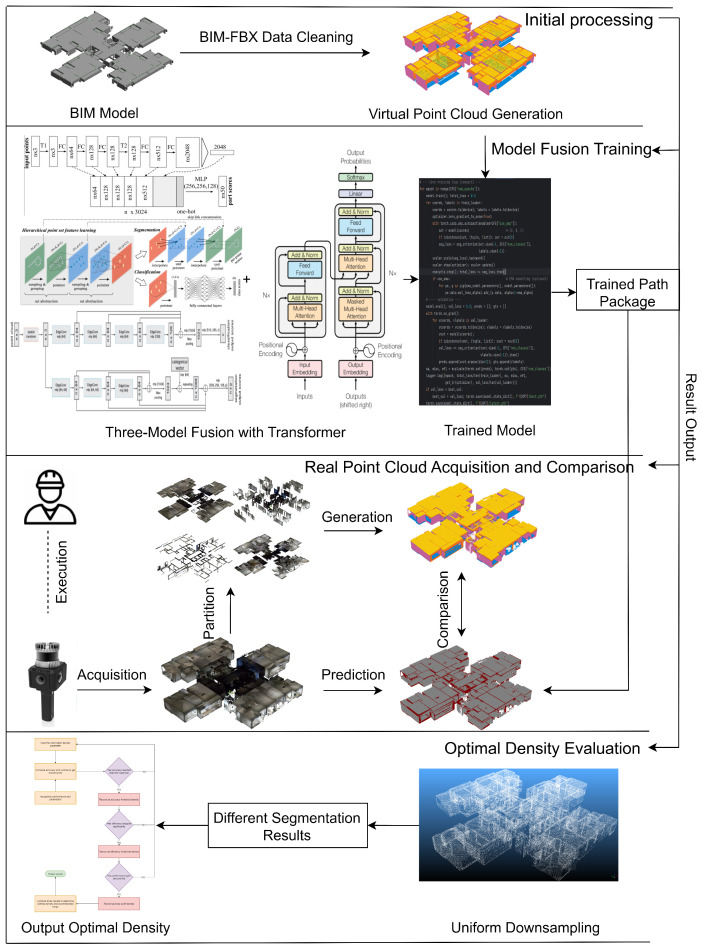
Overall workflow diagram.

By adopting a closed-loop design that integrates dual data sources, a unified model, and standardized density evaluation, we effectively isolate “sampling density” as the key independent variable, decoupling it from any model or training differences. Consequently, our framework yields optimal sampling density values and recommended parameter intervals for engineering deployment, enhancing both reproducibility and practical applicability. Concretely, the end-to-end workflow comprises: (i) generating virtual tiles from BIM (in parallel with cleaning/labeling real scans); (ii) tiling and normalizing inputs to a fixed point count for training; (iii) training three representative backbones with an identical Transformer module under one protocol; (iv) producing a series of uniformly down-sampled test densities via voxel+bisection; (v) sliding-window inference and metric aggregation; and (vi) applying the three-condition rule (accuracy sufficiency, efficiency decay, knee) to output the optimal density and an engineering-feasible range. The overall experimental workflow is illustrated in Figure 2.

### 3.2. Data Acquisition and Preprocessing

#### 3.2.1. Virtual Point-Cloud Processing

To address the issue of missing data and the difficulty of dense sampling in real-world scans, we employ virtual point clouds for deep-learning training [10,11]. Starting from the project’s as-built BIM model, we batch-export the model to FBX format, then preprocess and convert it into virtual point clouds to form a controllable, large-scale training set.

Because FBX models exported directly are solid and sometimes lack key elements (such as floor slabs), notable discrepancies can arise between the virtual and real point clouds. To enhance the realism and structural coherence of the virtual point clouds, we design an automated preprocessing pipeline in Blender with the following steps: (i) detect the lowest point of the scene, perform global alignment of the model, unify normals, and remove thin shell elements; (ii) apply component-specific filtering based on face orientation (retain vertical faces for walls and columns, and horizontal faces for slab components); (iii) for slab components, remove all non-horizontal faces; and (iv) complete the ground and floor slabs at the minimum column base elevation to improve consistency with real scenes. This strategy compensates for missing slab elements in the FBX and ensures overall spatial continuity. The detailed procedure is provided in Algorithm 1. In Algorithm 1, thickness_thr is the minimum element thickness (m) for removing paper-thin BIM artifacts, and angle_thr (°) limits facet orientation to suppress misoriented fragments before sampling.
**Algorithm 1:** Virtual point cloud preprocess from FBX **Input**: FBX models $\left\{M_{i}\right\}$; thresholds (thickness_thr,angle_thr) **Output**: Clean point clouds $\left\{P_{i}\right\}$ 
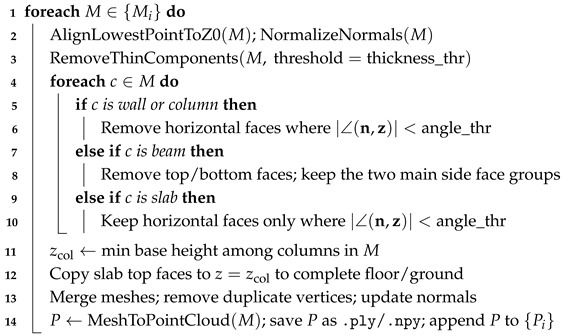


**Figure 3:**
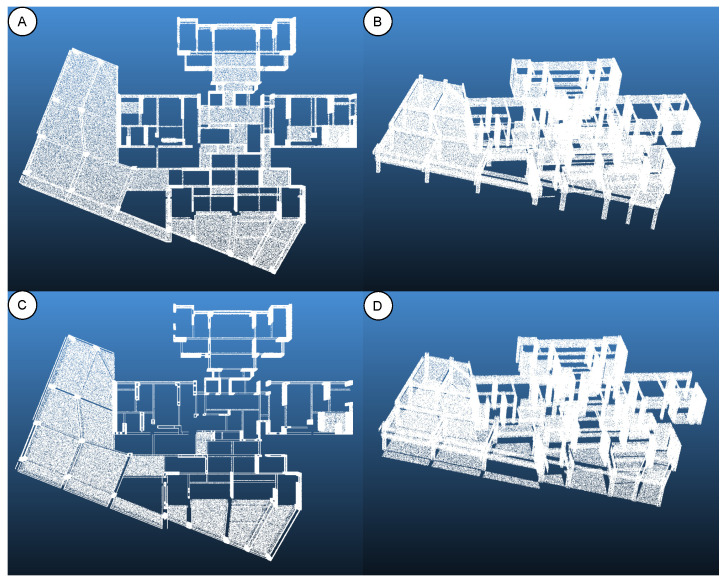
Comparison of virtual point cloud preprocessing (before vs. after). (**A**) Top view before; (**B**) oblique view before; (**C**) top view after; (**D**) oblique view after.

After preprocessing, the FBX model is converted from a solid into a hollow structure with completed floor slabs and ground, making the virtual point cloud distribution much closer to that of a real scan. Figure 3 illustrates this improvement: before processing (panels A, B), the model was internally closed and missing floor slabs, whereas after processing (panels C, D), the interior has been “hollowed out,” the slabs are completed, and structural continuity is greatly enhanced. The optimized FBX model is then converted into a high-density point cloud and divided into four component categories (walls and columns, beams, floor slabs, and ceilings), with unique component identifiers assigned to maintain correspondence between the point cloud and its BIM attributes. Finally, the point cloud is exported in .ply format and converted to .npy, providing standardized input data for the deep-learning models.

#### 3.2.2. Real Point-Cloud Acquisition and Processing

To evaluate the model’s adaptability and robustness in real engineering environments, we incorporate real laser-scanned point clouds collected from an active construction site as an independent test set. In addition, we establish a complete validation pipeline covering data acquisition, preprocessing, manual annotation, model inference, and final performance evaluation.

First, we deploy a mobile laser scanner on site to capture indoor point clouds from multiple viewpoints, taking advantage of the device’s flexible sampling capability. To ensure consistency and complete coverage, a multi-station scanning strategy is used during acquisition, and point clouds from different stations are precisely aligned using the Iterative Closest Point (ICP) registration algorithm [19]. This registration maintains continuity of the overall geometry and ensures global consistency. The raw point clouds are then preprocessed to suppress sensor noise and remove redundant points, improving clarity and usability. To align with engineering practice and common public-dataset preprocessing, we denoise, register, and uniformly down-sample the test point clouds so that density control and noise characteristics are reproducible, improving cross-project transferability and the auditability of decisions. Additionally, for the Scan-to-BIM workflow, we apply downsampling and quality control measures to reduce point count and computational cost while preserving geometric fidelity [18].

**Figure 4:**
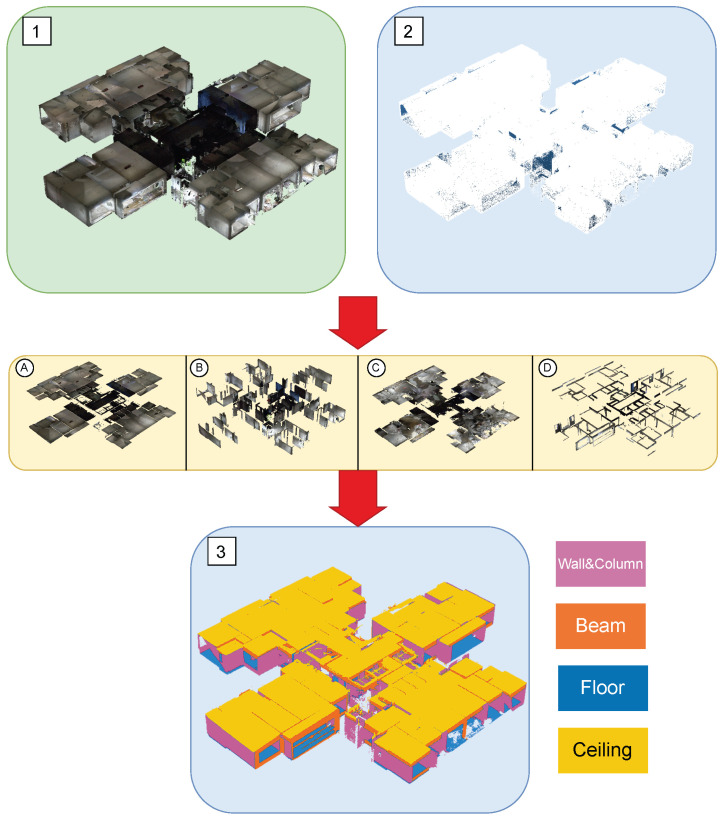
Schematic diagram of the real point cloud segmentation process ((**1**)—preprocessed color point cloud; (**2**)—preprocessed grayscale point cloud; (**3**)—semantic visualization of the final labeled point cloud; (**A**–**D**)—class-wise masks ((**A**), ceiling; (**B**), walls/columns; (**C**), floor; (**D**), beams)).

For ground-truth labeling, we use CloudCompare to manually segment the point clouds into four primary component classes: ceilings, walls and columns, floors, and beams. Figure 4 illustrates the annotation results. Panels (1) and (2) show the preprocessed point clouds in color and grayscale, respectively. Panel (3) presents the final semantic segmentation after multi-source data fusion, and panels A–D provide class-specific close-ups (A—ceiling; B—walls/columns; C—floor; D—beams). This manual labeling approach ensures high confidence in the dataset and maintains structural soundness under real-world conditions [6].

Finally, we convert the annotated point clouds into a standardized .npy format and feed them into the trained deep-learning model for inference. The model’s outputs are then compared point-by-point with the manual labels to quantitatively evaluate segmentation performance on the real point clouds, thereby confirming the model’s adaptability to complex building components.

### 3.3. Training Setup

#### 3.3.1. Model Architecture Overview

To prevent architecture-specific differences from confounding the “sampling density–performance” relationship and to examine the generality of our approach across different feature-modeling paradigms, we build a unified segmentation framework based on three classical backbones: PointNet–Trans, PointNet++–Trans, and DGCNN–Trans. Here, PointNet denotes a point-wise MLP with symmetric pooling for global feature aggregation, PointNet++ extends this with hierarchical set abstraction and local neighborhood grouping, and DGCNN encodes local topology using EdgeConv on dynamic k-nearest neighbors (k-NN) graphs. The overall model architecture is as follows: each backbone first extracts basic geometric features; a Point Transformer module (for global context aggregation) is then introduced to capture both local and global information [45]; channel-wise feature fusion and class-aware attention are applied for further refinement; and finally, a unified point-wise classification head outputs semantic probabilities for the four component categories (walls and columns, beams, floors, and ceilings) [42]. To ensure fair comparability, all three fusion pipelines use the same Transformer depth, hidden feature dimensionality, and classification head structure, and their training procedures and data preprocessing are fully aligned. This uniform configuration guarantees that any performance differences can be attributed solely to changes in the input sampling density.

To ensure broad coverage of common point-cloud feature learning strategies, we choose PointNet, PointNet++, and DGCNN as the representative backbones. PointNet uses shared MLP layers as a lightweight global feature extractor, which helps us observe the direct impact of density changes on overall representations. PointNet++ employs hierarchical sampling and local feature aggregation to capture multi-scale geometric structure, making it suitable for examining how sampling density affects local context information. DGCNN encodes neighborhood topology through EdgeConv, which enables the study of density sensitivity in local-structure learning [43,46,47]. We insert an identical Transformer module after the high-level features in all three backbones and perform feature fusion, allowing these different modeling mechanisms to accommodate the “density” variable in a consistent way. Accordingly, our analysis centers on quantifying the optimal sampling density rather than comparing the relative superiority of the models. To keep the density–performance relation interpretable and reproducible, we deliberately select three classical yet representative backbones that span point-wise MLPs (PointNet), hierarchical grouping (PointNet++), and graph-based local topology (DGCNN). Recent state-of-the-art systems such as sparse 3D CNNs (e.g., MinkowskiNet), efficient random sampling networks (e.g., RandLA-Net), and Transformer-only encoders (e.g., Point Transformer) employ architecture-specific sparsity or sampling policies that materially change runtime/memory scaling. Introducing them here would confound “density” with model capacity and framework-level optimizations. Our objective in this study is therefore to provide a model-agnostic selector where density is the only independent variable; systematic extensions to newer backbones under the same unified protocol are planned in follow-up work.

#### 3.3.2. Training Configuration and Implementation

To ensure that “sampling density” is the only variable under comparison in our experiments, we construct a unified and reproducible training pipeline on a single GPU. For different networks, only the local feature extraction branch is changed while all other training factors remain the same. All three fused models follow the same architecture paradigm—local encoding, Point Transformer–based global context, class-aware attention, and point-wise classification head—differing only in whether the local branch is PointNet, PointNet++, or DGCNN. Real point clouds are used solely as an external test set and are not included in training in order to avoid any distribution bias.

The training data consist of the tiled virtual point clouds prepared in Section 3.2. To eliminate any influence from varying input sizes, we use a custom collate_fn that pads or crops each sample to a fixed number of points *N*. In addition, automatic mixed precision (AMP), Exponential Moving Average (EMA) weight updates, learning rate scheduling, and early stopping are applied uniformly in all experiments [48].

We use Adam (initial learning rate $1\times 10^{-3}$, weight decay $5\times 10^{-4}$) as the optimizer, combined with a OneCycleLR learning rate schedule that first increases and then decreases the rate [49]. This “rise-then-fall” policy reduces oscillations in early training and accelerates convergence. An Exponential Moving Average (EMA) of the model weights is maintained to smooth out parameter updates and improve generalization [50], and the EMA weights are used for inference during validation and testing. This choice follows classical Polyak averaging, which is known to produce more stable parameter estimates in stochastic optimization  [51]. We set the maximum training epochs to 500 and enable Early Stopping with a patience of 10 epochs [52]. If the validation metrics show no significant improvement for 10 consecutive epochs, training is automatically terminated; conversely, if the model converges earlier, we stop training to avoid overfitting and reduce unnecessary computation.

Data augmentation is decoupled from sampling density and includes random rotation about the Z-axis, isotropic scaling, random translation, Gaussian jitter, small-percentage point dropping, and local occlusion. The augmentation magnitudes and probabilities are kept identical across all experiments and do not change the sampling density itself. The evaluation protocol is also consistent between training and validation: at each epoch we compute Overall Accuracy (OA), mean Intersection-over-Union (mIoU), macro F1-score, and per-class IoU, and we generate a confusion matrix for error analysis. Here, OA denotes the fraction of correctly classified points; mIoU is the mean of class-wise intersection-over-union; macro F1-score is the unweighted average of per-class F1; and IoU is defined as TP/(TP+FP+FN). The best model weights are saved based on validation performance, and Early Stopping is triggered as described above if applicable. The parameter configurations for training and evaluation are summarized in Table 1. We adopt Adam for adaptive moment estimation with per-parameter step sizes and bias-corrected first/second moments, a widely validated choice for deep architectures in practice  [53]. For clarity, AMP denotes automatic mixed precision to reduce memory use and improve throughput, and EMA denotes the exponential moving average of model weights for more stable evaluation.

To keep sampling density as the sole comparative variable, we apply automatic mixed precision (AMP), exponential moving average (EMA) of weights, a single learning–rate schedule, and early stopping uniformly across all experiments. AMP reduces GPU memory and wall-time at the same batch size while preserving numerical stability via dynamic loss scaling; EMA yields lower-variance validation estimates under identical data and training length; a single schedule (OneCycleLR with the same maximum learning rate as in Table 1) harmonizes convergence behavior across backbones; and early stopping (patience = 10 on validation metrics) prevents over-training from confounding density effects. These controls standardize optimization and evaluation so that any observed differences arise from density rather than training heuristics.

For the loss function, a point-wise weighted cross-entropy is adopted to mitigate class imbalance among components. It is defined as

(1) $$L_{\mathrm{seg}}=-\frac{1}{N}\sum_{i=1}^{N}\sum_{c=1}^{C}w_{c}\;y_{i,c}\;\log p_{i,c},$$

where $y_{i,c}$ denotes the one-hot label, $p_{i,c}$ the predicted probability, and $w_{c}$ the class weight. With label smoothing enabled $\left(\epsilon \in [0,1)\right)$, the labels are rewritten as

(2) $${\tilde{y}}_{i,c}\;=\;\left(1-\epsilon \right)\;y_{i,c}\;+\;\frac{\epsilon }{C}.$$

The Exponential Moving Average weights are updated by

(3) $$\theta_{\mathrm{EMA}}\;\leftarrow \;\beta \;\theta_{\mathrm{EMA}}\;+\;\left(1-\beta \right)\;\theta .$$

Here, β∈(0,1) controls the averaging window (larger β gives heavier smoothing and slower adaptation).

During validation and testing, EMA weights are used for inference to obtain more stable evaluations. At this point, all non-density factors in training have been unified, ensuring that density remains the only comparative variable in subsequent experiments; the concrete implementation is provided in Algorithm 2.
**Algorithm 2:** Training pipeline for point-cloud segmentation. **Input**: Virtual point-cloud dataset D (blocks), model M∈{PointNet–Trans, PointNet++–Trans, DGCNN–Trans}, epochs *E*, fixed point number *N* **Output**: Best weights $\theta^{★}$ (EMA preferred) 
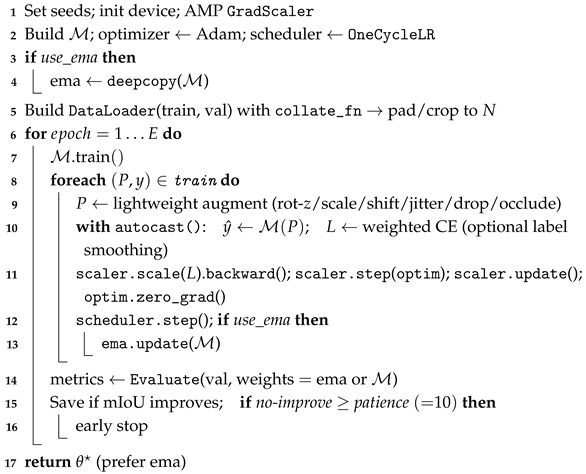


Training and evaluation settings are kept identical across all density levels: per-tile normalized points N=8192; batch size B=32; maximum epochs E=500 with early stopping (patience =10); Adam optimizer (initial learning rate $1\times 10^{-3}$, weight decay $5\times 10^{-4}$) with OneCycleLR; AMP and EMA enabled; augmentations include rotation about the *Z* axis, isotropic scaling, translation, Gaussian jitter, and sparse point dropping; validation metrics: OA, mIoU, and macro-F1.

In our DataLoader, the collate function standardizes each sample to a fixed budget *N* points: if a tile exceeds *N*, points are randomly subsampled; if smaller, we pad coordinates/features to *N* and set the extra labels to an ignore_label. During training we also apply lightweight augmentations (rotation about *Z*, isotropic scaling, translation, jitter, sparse point dropping) within the collate stage, whereas validation only pads. The resulting batch tensors have shapes (B,N,C) for coordinates/features and (B,N) for labels, ensuring that batching effects do not confound density.

After training, the model is applied to inference on real point clouds. In the testing phase, we reuse the same tiling and overlap strategy as in training, segmenting the input point cloud block by block and performing voting fusion in overlapping regions to reconstruct complete component-level semantic labels. The results are then compared with those from synthetic data to assess recognition performance across data sources. The classification system includes four component categories—walls and columns (0), beams (1), floors (2), and ceilings (3)—and we compute category-wise accuracy and coverage to analyze class-level differences.

The evaluation metrics remain consistent with those used during training and validation: at each epoch we compute Overall Accuracy (OA), mean Intersection over Union (mIoU), macro-F1, and per-class IoU, and generate a confusion matrix for error diagnosis. IoU measures the overlap between predictions and ground truth; mIoU is the arithmetic mean of per-class IoUs; F1 combines precision and recall to characterize overall classification effectiveness.

To ensure comparability and reproducibility, the three fused models use exactly the same settings for batch size, maximum epochs, augmentation magnitude, optimization and scheduling, Early Stopping, EMA, and the evaluation protocol [6].

### 3.4. Optimal Density Criterion

#### 3.4.1. Uniform Downsampling

Point-cloud sampling density is a key factor influencing semantic segmentation accuracy and reconstruction quality, and balancing geometric fidelity against computational efficiency has long been a central issue in point cloud analysis. To isolate “sampling density” as the sole variable in our experiments, we adopt a uniform downsampling strategy during evaluation so that input conditions remain consistent across all comparison groups.

In our implementation, the point cloud is partitioned with a 3D voxel grid. Given a raw point cloud and a target point count *T*, we perform a binary search on the voxel size *s* to downsample the cloud to approximately *T* points. At each iteration, one representative point is retained per voxel, and the resulting point count is compared with *T*; if the sampled count exceeds *T*, the voxel size is increased, otherwise it is decreased. This process iteratively adjusts *s* until the point count is within a specified tolerance of *T* (or a maximum number of iterations is reached). By repeating this procedure for a series of target counts $\left\{T_{1},T_{2},\ldots ,T_{K}\right\}$, we generate multiple density levels, enabling density–performance curve analysis. (Pseudocode for this method is provided in Algorithm 3).

In Algorithm 3, [L,U] are the bisection bounds for voxel size (m), ε is the relative tolerance to the target count, $\left\{T_{k}\right\}$ are desired totals per density, and *s* is the current voxel size under evaluation.

Uniform voxel down-sampling with total-point control uses a bisection search on the isotropic voxel size *s*: given a target total per tile $\left\{T_{k}\right\}$ (or target surface densities $\left\{\delta_{k}\right\}$), search s∈[L,U] derived from scene scale and stop when $\left|\;|Q\right|-T_{k}\;|\leq \varepsilon$; the grid is anchored in the tile frame for reproducibility and density is calibrated against the floor area $A_{\mathrm{total}}$.

**Figure 5:**
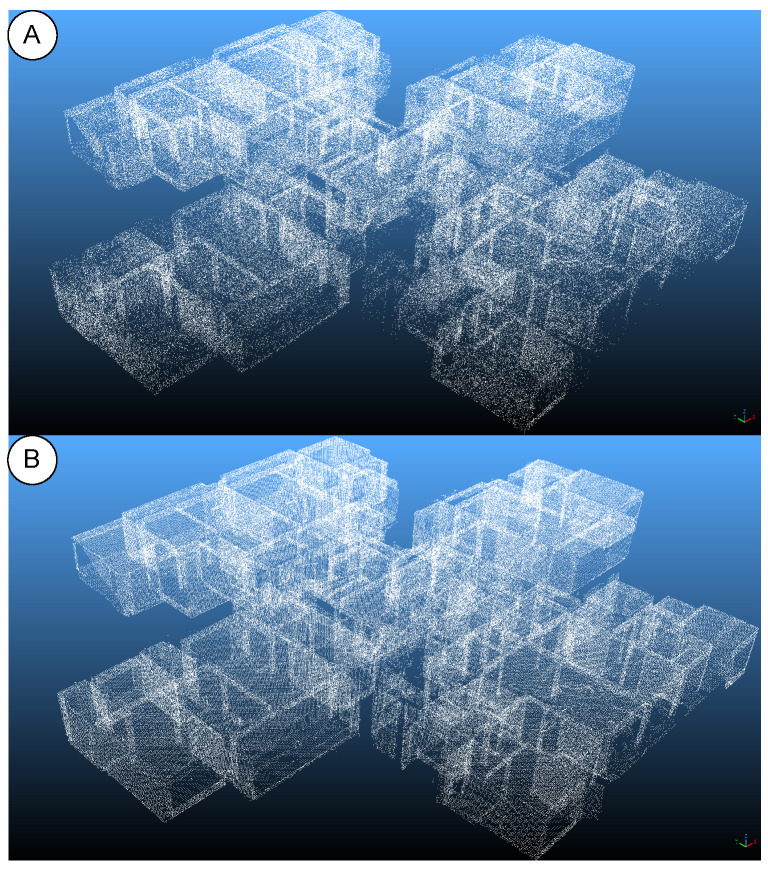
Comparison of sampling methods. (**A**) Random sampling; (**B**) uniform sampling.

Compared to random downsampling, the uniform method distributes points more evenly in 3D space and is highly reproducible. It reduces the point count while preserving the overall geometric skeleton and primary structural features of the scene. Figure 5 highlights the difference: random sampling (panel A) tends to create local gaps and uneven point density, whereas uniform sampling (panel B) produces a more regular and stable result that better meets the experimental requirements [20,22,25].
**Algorithm 3:** Uniform grid downsampling with total-point control. **Input**: Point cloud *P* (or directory), target totals $\left\{T_{1},\ldots ,T_{K}\right\}$, voxel bounds [L,U], tolerance ε **Output**: $\left\{P_{T_{k}}^{*}\right\}$ with $\left|P_{T_{k}}^{*}\right|\approx T_{k}$; PLY per target 
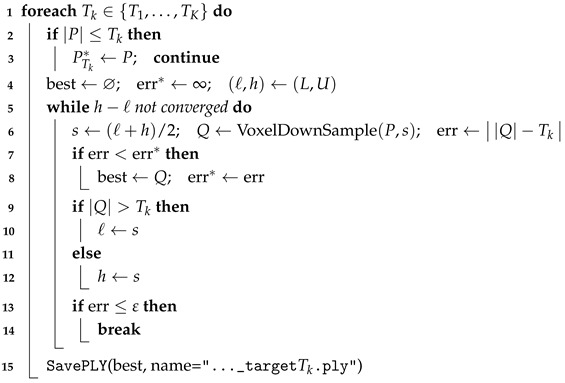


#### 3.4.2. Optimal Density Determination

To determine the optimal sampling density for the segmentation model without altering the training and evaluation protocols, a monotonically increasing density sequence $\left\{\delta_{i}\right\}$ (points/m^2^) is generated on a unified high-density raw point cloud. The same inference procedure is executed on each of the three fused models, and the corresponding recognition performance and processing time are recorded. Density is calibrated against the total surface area of the test floor, $A_{\mathrm{total}}$, by

(4) $$\delta_{i}\;\approx \;\frac{\left|P_{i}\right|}{A_{\mathrm{total}}}\;,$$

where $\left|P_{i}\right|$ is the total number of points for the *i*-th density version. To reduce randomness introduced by interval choices, $\left\{\delta_{i}\right\}$ is designed to be equally spaced, and measurements are repeated at each density level to smooth fluctuations.

To characterize overall performance as a function of density, we define a composite score G(δ) that combines accuracy and coverage; we refer to G(δ) as the weighted accuracy (WA). In particular, we instantiate *G* as a weighted average of overall accuracy and overall IoU:

(5) $$G\left(\delta_{i}\right)\;=\;\frac{2\;\mathrm{Acc}\left(\delta_{i}\right)+\mathrm{IoU}\left(\delta_{i}\right)}{3}\;.$$

Here Acc denotes OA and IoU denotes mIoU; the 2:1 weighting emphasizes overall correctness while still accounting for class-overlap quality.

We also define a marginal gain with respect to density [2] to quantify the incremental improvement from $\delta_{i-1}$ to $\delta_{i}$:

(6) $$\Delta G_{i}\;=\;\frac{G\left(\delta_{i}\right)-G\left(\delta_{i-1}\right)}{\delta_{i}-\delta_{i-1}}\;,\;i\geq 2\;.$$

The processing time at each sampling level, $T(\delta_{i})$, is then incorporated, together with a marginal-efficiency index that quantifies accuracy gain per unit time:

(7) $$\Delta T_{i}\;=\;\frac{T\left(\delta_{i}\right)-T\left(\delta_{i-1}\right)}{\delta_{i}-\delta_{i-1}},\;i\geq 2.$$

(8) $$\eta_{i}\;=\;\frac{\Delta G_{i}}{\Delta T_{i}}.$$

T(δ) is the end-to-end wall-clock time (in seconds), so $\eta_{i}$ measures accuracy gain per unit time between two adjacent densities.

This represents the accuracy gain per unit time when increasing density from $\delta_{i-1}$ to $\delta_{i}$.

To eliminate subjective thresholds, we determine the optimal density using three quantitative criteria evaluated by a script: “accuracy sufficiency,” “efficiency decay,” and “knee point.” Specifically, let $G_{\max}=\max_{i}G\left(\delta_{i}\right)$. The accuracy-sufficiency point $\delta_{\alpha }$ is defined as the lowest density at which $G\left(\delta \right)\geq 0.98\;G_{\max}$, meaning any further densification would yield less than a 2% performance gain. Next, let $\eta_{\max}=\max_{i}\eta_{i}$. The efficiency-decay point $\delta_{\beta }$ is identified when $\eta_{i}$ drops to 10% of $\eta_{\max}$ (or lower) for two consecutive density increments; the rightmost density in that low-efficiency span is taken as $\delta_{\beta }$, indicating that beyond this point the accuracy return per unit time has fallen to a very low level. Finally, the knee point $\delta_{\mathrm{knee}}$ marks where the *G*–density curve transitions from a steep increase to a gradual increase. We define $\delta_{\mathrm{knee}}$ as the first density at which the slope $s_{i}=\Delta G_{i}$ decreases to ≤25% of the maximum slope observed.

For a given model, we combine these criteria to obtain a single optimal density. We select a conservative value:

(9) $$\delta_{\mathrm{opt}}\;=\;\max\left\{\delta_{\alpha },\;\delta_{\beta },\;\delta_{\mathrm{knee}}\right\}\;.$$

We use α for accuracy sufficiency, β for relative efficiency-decay, and γ for knee sensitivity (with smoothing radius *r*); $\delta_{\mathrm{opt}}$ conservatively takes the rightmost threshold among the three.

The rightmost threshold is selected so that “accuracy is sufficient, efficiency is no longer economical, and the curve has turned.” To reflect uncertainty from discrete sampling, a stability interval $\left[\delta_{\alpha },\delta_{\beta }\right]$ is also reported; if crossings with $\delta <\delta_{\alpha }$ occur, $\left[\delta_{\mathrm{knee}},\delta_{\mathrm{opt}}\right]$ is used as an alternative.

Applying the above to the three fused models yields $\left\{\delta_{\alpha }^{\left(m\right)},\delta_{\beta }^{\left(m\right)},\delta_{\mathrm{knee}}^{\left(m\right)},\delta_{\mathrm{opt}}^{\left(m\right)}\right\}$. On this basis, a cross-model consensus is further provided:

(10a) $$\begin{array}{cc} \delta_{L}\; & =\;\max_{m}\;\delta_{\alpha }^{\left(m\right)}\;, \end{array}$$

(10b) $$\begin{array}{cc} \delta_{U}\; & =\;\min_{m}\;\delta_{\beta }^{\left(m\right)}\;, \end{array}$$

(10c) $$\begin{array}{cc} \delta^{*}\; & =\;{\mathrm{median}}_{m}\;\delta_{\mathrm{opt}}^{\left(m\right)}\;. \end{array}$$

Here *m* indexes the backbones; $\left[\delta_{L},\delta_{U}\right]$ is the cross-model feasible band and $\delta^{*}$ is the single-point recommendation.

**Figure 6:**
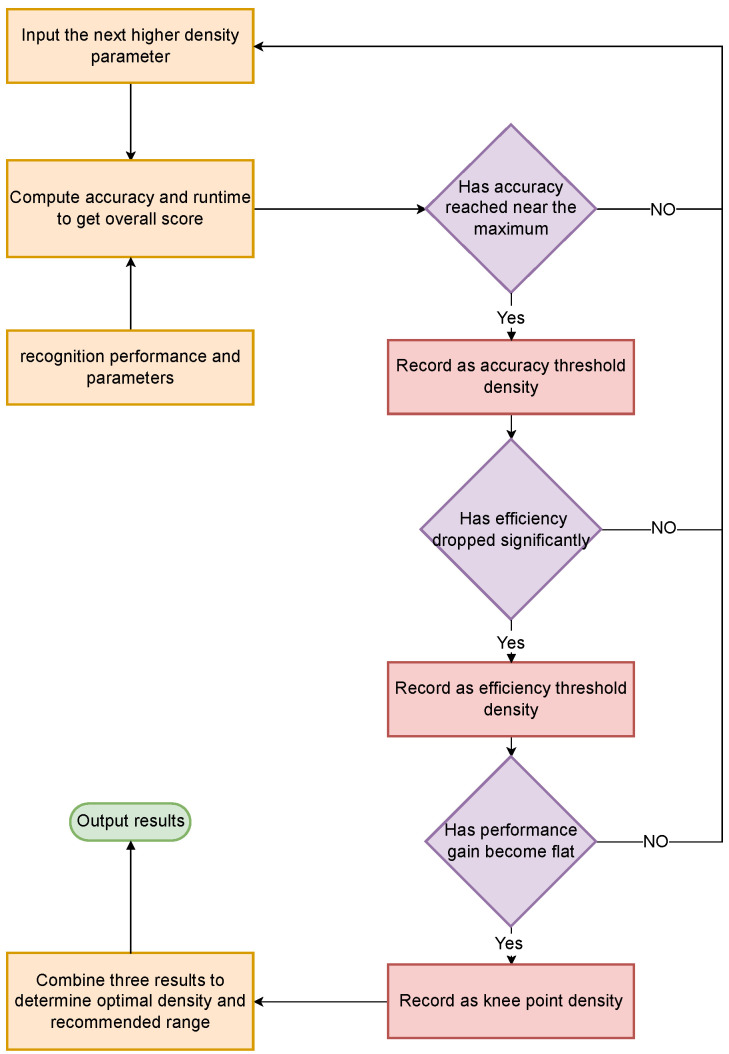
Flowchart of optimal sampling density selection.

When $\delta_{L}\leq \delta_{U}$, $\left[\delta_{L},\delta_{U}\right]$ is the density range jointly endorsed by the three models, and $\delta^{*}$ serves as a single-point recommendation for engineering. The overall implementation flow is shown in Figure 6.

For reproducibility, we implement the above criteria in a procedural script. The discrete data tuples $\left(\delta_{i},G_{i},T_{i}\right)$ obtained from the experiments serve as inputs. We apply a slight smoothing to the *G* values and compute finite differences to obtain $\Delta G_{i}$, $\Delta T_{i}$, and $\eta_{i}$. The script then automatically finds $\delta_{\alpha }$, $\delta_{\beta }$, and $\delta_{\mathrm{knee}}$, computes $\delta_{\mathrm{opt}}=\max\left\{\delta_{\alpha },\delta_{\beta },\delta_{\mathrm{knee}}\right\}$, and reports the stability interval along with a summary of the key results. Algorithm 4 provides the pseudocode for this optimal-density selection procedure.

The optimal-density decision rule depends on four scalars: accuracy sufficiency α (e.g., 0.98), relative efficiency-decay β (e.g., 0.10), smoothing radius *r* over adjacent density levels (e.g., 2), and knee-sensitivity γ (e.g., 0.25 of the maximum slope); unless stated otherwise, these defaults are used throughout.
**Algorithm 4:** Optimal sampling density selection (code-implemented).**Input**: Ordered tuples ${\left\{\left(\delta_{i},G_{i},T_{i}\right)\right\}}_{i=1}^{n}$; parameters α,β,r,γ**Output**: Per-model $\left\{\delta_{\alpha },\;\delta_{\beta },\;\delta_{\mathrm{knee}},\;\delta_{\mathrm{opt}}\right\}$; consensus $\left[\delta_{L},\;\delta_{U}\right]$, $\delta^{*}$**_1_** Sort tuples by δ; apply a small moving-average smoothing to *G***_2_** Compute discrete derivatives:   $dG_{i}\leftarrow \frac{G_{i}-G_{i-1}}{\delta_{i}-\delta_{i-1}}$,   $dT_{i}\leftarrow \frac{T_{i}-T_{i-1}}{\delta_{i}-\delta_{i-1}}$,   $\eta_{i}\leftarrow \frac{dG_{i}}{dT_{i}}$**_3_** $\delta_{\alpha }\leftarrow$ first δ with G(δ)≥α·max(G)**_4_** $\delta_{\beta }\leftarrow$ right end of the first run of *r* densities with η≤β·max(η)**_5_** $\delta_{\mathrm{knee}}\leftarrow$ first δ with dG≤γ·max(dG)   (fast→slow gain turning)**_6_** $\delta_{\mathrm{opt}}\leftarrow \max\left\{\delta_{\alpha },\;\delta_{\beta },\;\delta_{\mathrm{knee}}\right\}$      (conservative pick)**_7_** (Optional per-model band) stable interval: $\left[\min\left(\delta_{\alpha },\delta_{\beta }\right),\;\max\left(\delta_{\alpha },\delta_{\beta }\right)\right]$**_8_** Across models:   $\delta_{L}\leftarrow \max_{m}\;\delta_{\alpha }^{\left(m\right)}$;   $\delta_{U}\leftarrow \min_{m}\;\delta_{\beta }^{\left(m\right)}$;   $\delta^{*}\leftarrow {\mathrm{median}}_{m}\;\delta_{\mathrm{opt}}^{\left(m\right)}$**_9_** Export plots: *G*–δ with α/β/knee markers (blue/red/green) and $\delta_{\mathrm{opt}}$ (black); η–δ with β and $\delta_{\mathrm{opt}}$**_10_** Export CSV/JSON of curves and markers for reproducible plotting

## 4. Experiments and Results

### 4.1. Site Description

We selected a large residential building project in Guangzhou, Guangdong Province, as the study site. The project includes several high-rise residential towers, a basement, and other facilities, with Building No. 10 chosen as the primary subject of analysis. This building rises 32 stories, features a complex structural system, and contains multiple apartment layouts. The on-site space is constrained and working conditions are difficult, representing a typically challenging construction scenario. Notably, deploying LiDAR point–cloud scanning for construction acceptance (e.g., as part of a Scan-to-BIM validation workflow) is feasible at this site, making it a suitable setting for our experiments. The selected standard-floor scene exhibits representative structural complexity, occlusions, and access constraints typical of indoor construction, making a single-case evaluation adequate to reveal the practical density–efficiency response.

### 4.2. Experimental Setup

In our experiments, on-site color point clouds were captured with a Lingjing MetaCam 3D laser scanner (Wuqiong Innovation). This device is compact (113 × 122 × 295 mm) and lightweight (1.57 kg), can scan up to 650,000 points per second, and includes a 12.3-megapixel HD camera for imaging. It has a maximum range of 120 m and offers roughly 1 h of battery operation, which makes it well-suited for mobile surveying in complex construction environments. Its portability and high throughput allow efficient capture of detailed point clouds in the field.

For training data preparation, we generated synthetic point-cloud tiles from the Building No. 10 BIM model, with each point labeled by its semantic class. These “virtual” point clouds provided large-scale, labeled training samples. The experimental platform consists of an NVIDIA GeForce RTX 3080 Ti GPU and an Intel Xeon Gold 6266C CPU. To maintain independence between training and testing distributions, floors B1 and 10–30 were used for model training/validation, while floors 5–9 served as the test set. In total, over 50,000 training sub-tiles were constructed, each standardized to a fixed number of points. Table 2 summarizes the data sources and floor splits.

During model training, all three fused models (each backbone with a Transformer block) were run under a unified configuration: a batch size of 32 and an initial learning rate of 0.001. Although training was allowed to run for up to 500 epochs, the models actually converged in fewer iterations. PointNet++–Trans stabilized by about 50–80 epochs, DGCNN–Trans by about 100–150 epochs, and PointNet–Trans only plateaued around 150–200 epochs. An early stopping criterion was therefore applied to terminate training once convergence was observed, avoiding redundant computation and thus saving training time and energy while maintaining good generalization.

**Figure 7:**
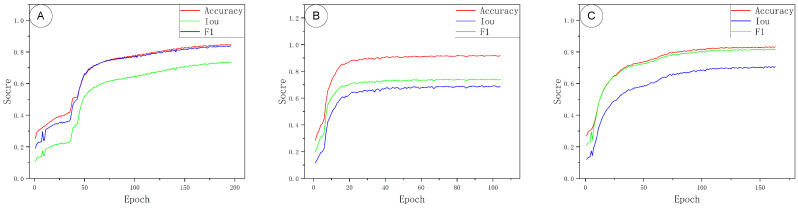
Model evaluation metrics curves. (**A**) PointNet–Trans; (**B**) PointNet++–Trans; (**C**) DGCNN–Trans.

The training progress of the three models is illustrated in Figure 7. In panels A, B, C (PointNet–Trans, PointNet++–Trans, DGCNN–Trans, respectively), Accuracy, IoU, and $F_{1}$-score all rise rapidly in the initial epochs. PointNet–Trans required roughly 150–200 epochs to stabilize all three metrics. PointNet++–Trans converged much sooner (within ∼50–80 epochs). DGCNN–Trans showed an intermediate pattern: Accuracy and $F_{1}$ stabilized by ∼40–70 epochs, whereas IoU continued improving until about 120–160 epochs.

**Figure 8:**
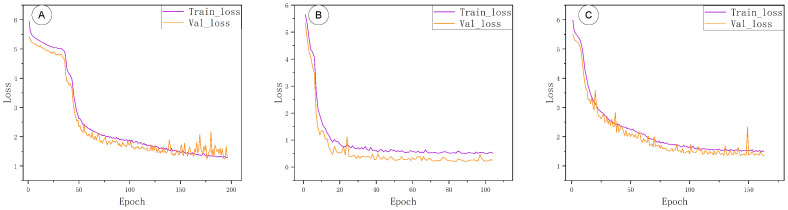
Training and validation loss curves. (**A**) PointNet–Trans; (**B**) PointNet++–Trans; (**C**) DGCNN–Trans.

Figure 8 shows the training-loss and validation-loss curves for the same three models (panels A, B, C). All models’ losses dropped sharply within the first 30–60 epochs and then flattened at low values. The validation-loss curves exhibit only minor fluctuations, indicating that training largely converged without significant overfitting.

To evaluate the model’s engineering applicability, we conducted an on-site scan of a standard floor (the third floor, with a surface area of $4511\;m^{2}$) that had clearly defined construction milestones. This full-floor LiDAR scan yielded over $5.88\times 10^{7}$ points and provided comprehensive coverage of key structural components such as beams, walls, columns, and slabs. The raw point cloud was subsequently filtered, registered, and colorized. The processed point-cloud model was then fed into the trained network for semantic inference and classification, simulating an actual deployment of the segmentation system on a construction floor.

### 4.3. Analysis of Experimental Results

#### 4.3.1. Segmentation on Real Point Clouds

Under the unified evaluation protocol, the full-floor point cloud was partitioned into overlapping sub-tiles using a sliding window approach. Any excessively large tiles (above a set point-count threshold) were further split to ensure manageable sub-tile sizes. After performing semantic segmentation on each sub-tile, we exported the predicted labels (as .npy files) and the visualization outputs (as .ply files), and we recorded the processing time for throughput analysis.

**Figure 9:**
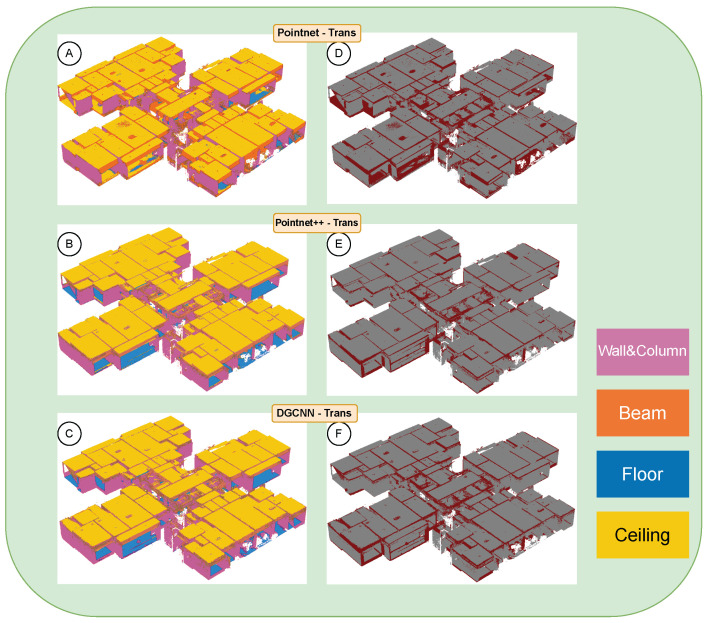
Semantic segmentation results on the real building. (**A**–**C**) Predictions by PointNet–Trans, PointNet++–Trans, and DGCNN–Trans; (**D**–**F**) error overlays—correct regions in gray and misclassified points in dark red.

Figure 9 demonstrates the semantic segmentation results of the three models on the high-density real point cloud. The left column (panels A–C) shows the semantic predictions by PointNet–Trans, PointNet++–Trans, and DGCNN–Trans, respectively, while the right column (panels D–F) presents the error overlays (gray indicates correctly classified regions and red indicates misclassified points). We use a consistent color scheme for all classes: walls and columns in magenta, beams in orange, floors in blue, and ceilings in amber yellow. Misclassification errors occur primarily at beam–wall junctions, in occlusion shadow regions, and along thin-walled elements, whereas broad planar areas are classified much more reliably [36]. These observations align with previous findings that smaller or more slender structural components require a higher point density and stronger contextual information to be recognized correctly [39,42].

The detailed performance metrics for each model and class are reported in Table 3, Table 4 and Table 5. PointNet–Trans (baseline) achieves an overall accuracy (OA) of 0.771 with a mean IoU of 0.565. PointNet++–Trans improves to an OA of 0.856 and mIoU of 0.732, with particularly high IoUs on planar components (e.g., Floor 0.903, Ceiling 0.841). DGCNN–Trans reaches a similar overall level (OA 0.854, mIoU 0.723) and shows a notable advantage on the beam class (Beam IoU 0.420 and Precision 0.559). Notably, for PointNet–Trans, floor segmentation exhibits a case of “high precision but low recall”—the Floor IoU is only 0.393 despite a Precision of 0.989—indicating that many floor points were missed (especially along boundaries and in occluded areas) even though the predictions it did make were nearly all correct. This gap suggests insufficient coverage of floor regions by the baseline model, likely due to its limited sensitivity in edge areas.

#### 4.3.2. Visualization Under Different Densities

For the sampling-density evaluation, we used only real site-scanned point clouds to ensure that conclusions are directly applicable to practical field conditions. Under a consistent evaluation protocol, we varied only the input point-cloud density while keeping all other processing steps identical. Using the uniform downsampling method described in Section 3.4.1, we generated 50 test samples with point densities ranging from 100 to 5000 points/m^2^. Each of the three fused models was then independently run on these samples for inference and evaluation.

**Figure 10:**
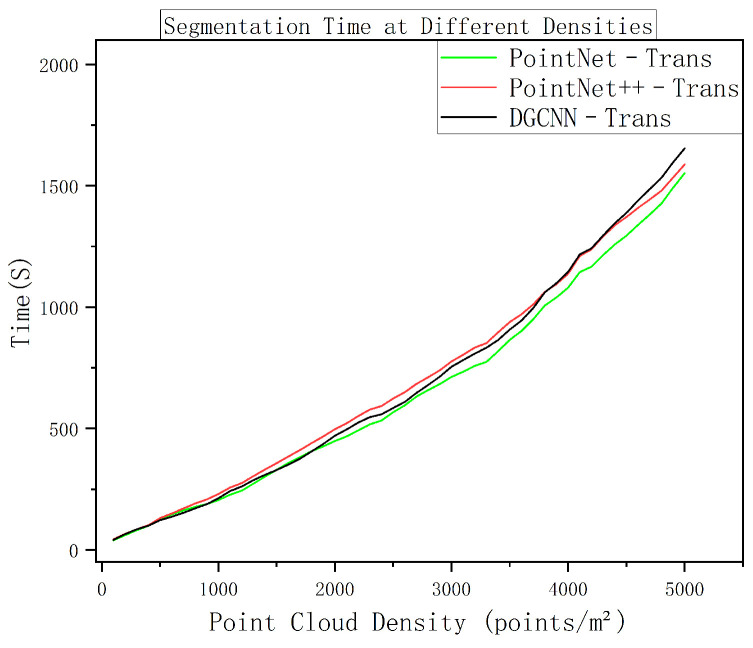
Inference time versus sampling density (three models in one plot).

Figure 10 plots the inference time versus sampling density for the three models. The total processing time increases roughly linearly as density grows, with only a slight acceleration observed at the highest densities. Notably, the timing curves of PointNet–Trans, PointNet++–Trans, and DGCNN–Trans almost completely overlap, indicating negligible differences in their runtime performance across the density spectrum [20,22]. This near-identical scaling behavior is consistent with prior reports on efficient large-scale point-cloud networks [54], and confirms that all three models are similarly impacted by increased data size.

**Figure 11:**
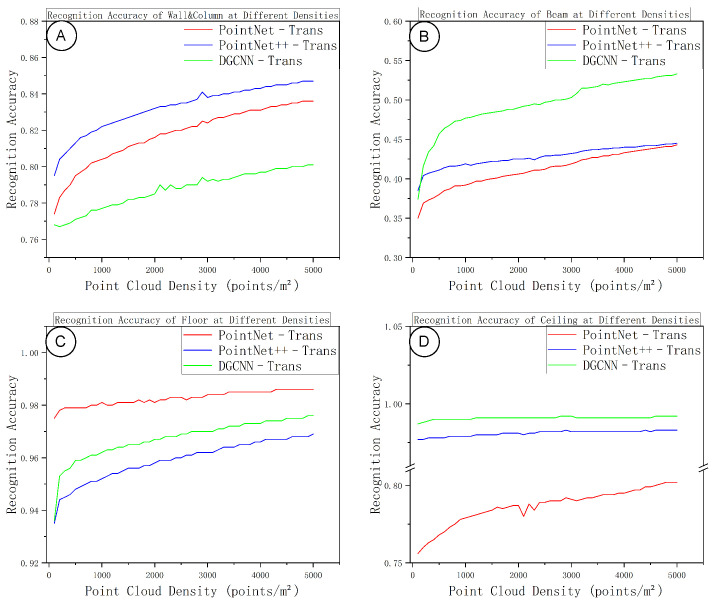
Per-class accuracy versus sampling density. (**A**) Walls/columns; (**B**) beams; (**C**) floors; (**D**) ceilings.

**Figure 12:**
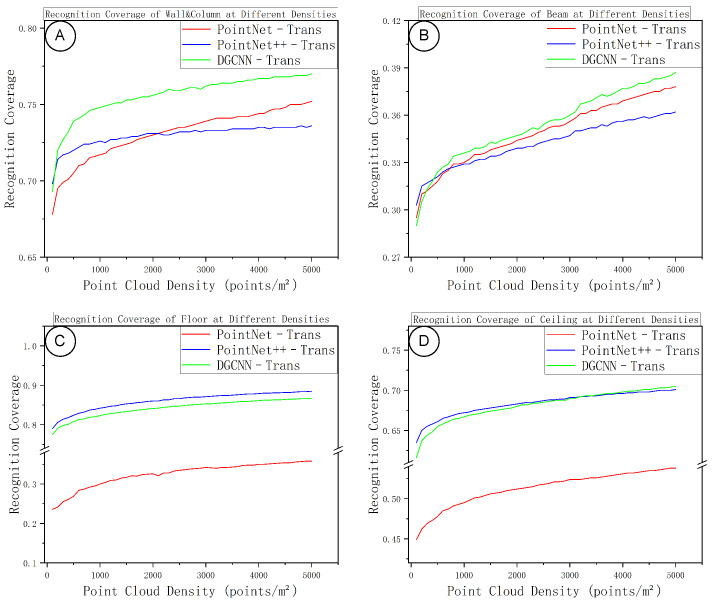
Per-class IoU versus sampling density. (**A**) Walls/columns; (**B**) beams; (**C**) floors; (**D**) ceilings.

Figure 11 and Figure 12 summarize the class-wise segmentation performance as a function of sampling density. In general, as density increases, the accuracy and IoU for all four component classes improve initially and then level off, forming a performance plateau at medium-to-high densities. Planar components (floors and ceilings) reach their peak accuracy at relatively lower densities, showing minimal gains from further densification beyond a moderate density. Walls and columns require somewhat higher point density than planar elements to stabilize, with their performance curve lying between those of planar elements and beams. Beams are the most sensitive class with respect to density: at low densities, the lack of continuous surface points and insufficient neighborhood context lead to frequent misclassifications; as density increases, the local neighborhood structure becomes more complete, the IoU for beams steadily improves, and the “performance knee” for beams occurs at a higher density compared to the other classes.

With regard to model differences, PointNet++–Trans is particularly robust on planar components, reaching its accuracy plateau earlier and with smaller fluctuations than the others. DGCNN–Trans shows a clear advantage for the beam class, achieving higher IoUs for beams thanks to more effective edge-feature extraction at medium and high densities. PointNet–Trans is slightly weaker overall than the other two models, yet it achieves the highest beam accuracy, indicating more conservative predictions with fewer false positives on slender elements (albeit missing some true positives at lower densities). At very high densities, the performance curves of all three models converge, and their relative rankings remain consistent from the medium-density range—implying that each model’s strengths and weaknesses persist even as density increases, but the gap between models does not widen further in the high-density regime.

**Figure 13:**
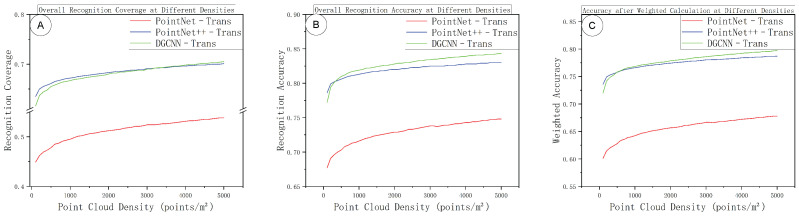
Overall performance versus sampling density: (**A**) overall accuracy (OA); (**B**) mean IoU (mIoU); (**C**) weighted accuracy (WA).

Figure 13 shows the overall segmentation performance metrics (Overall Accuracy, mean IoU, and weighted accuracy) as functions of sampling density. All these metrics improve with increasing density and gradually approach a ceiling at high densities. Across the board, PointNet++–Trans and DGCNN–Trans achieve very similar overall performance, and both consistently outperform PointNet–Trans at comparable densities. Considering also the near-linear runtime growth from Figure 10, these results indicate that the best accuracy–efficiency trade-off lies in the medium-density range. Pushing density higher yields only marginal accuracy gains, while computational load (memory usage and inference time) rises substantially. In other words, beyond a certain point, additional scan density gives diminishing returns in accuracy but incurs a steep penalty in processing cost, which is undesirable for practical deployment.

**Figure 14:**
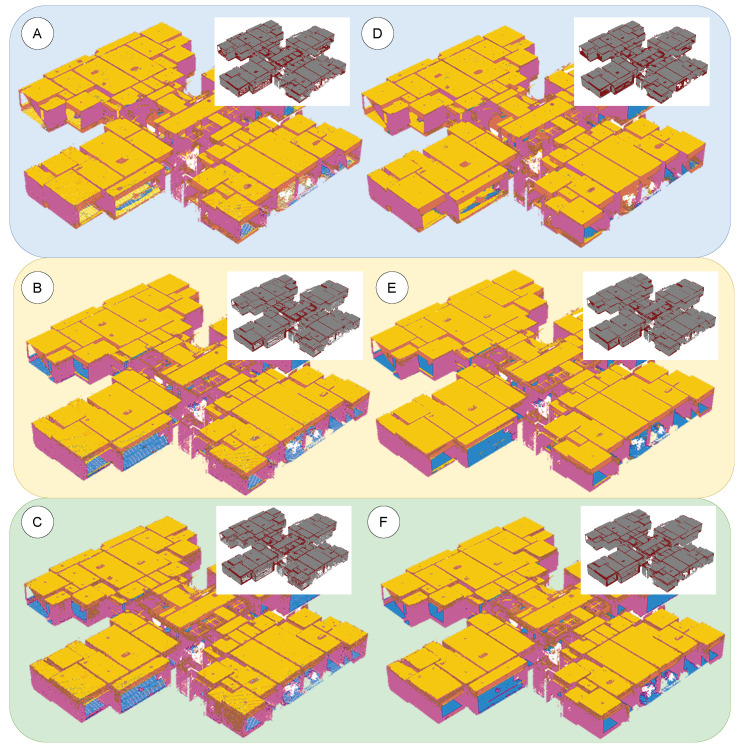
Segmentation results under high vs. low densities. (**A**–**C**) Low density for the three models; (**D**–**F**) high density for the three models; error overlays in dark red.

To further illustrate the effect of sampling density on segmentation outcomes, we visually compared model outputs at a low density (200 points/m^2^) versus a high density (5000 points/m^2^), as shown in Figure 14. The left column shows the low-density results for PointNet–Trans (A), PointNet++–Trans (B), and DGCNN–Trans (C), while the right column shows the corresponding high-density results for the same models (D, E, F). In each sub-figure, the large panel displays the color-coded semantic segmentation, and the small inset in the upper-right highlights the spatial distribution of correctly classified points (gray) versus misclassified points (dark red).

Across all three models, an excessively low sampling density leads to fragmented and noisy segmentation in certain regions. As the density increases, the segmentation results become more complete and accurate, though with diminishing improvement once a moderate density is surpassed. Notably, the difference between the very high (5000 points/m^2^) case and the unthinned, original-density point cloud is not significant; the 5000 points/m^2^ segmentation is statistically comparable to the full-density result. This confirms that, for indoor point-cloud segmentation, a moderately low sampling density is already sufficient to achieve the required accuracy in practice [4].

#### 4.3.3. Optimal Sampling Density Analysis

**Figure 15:**
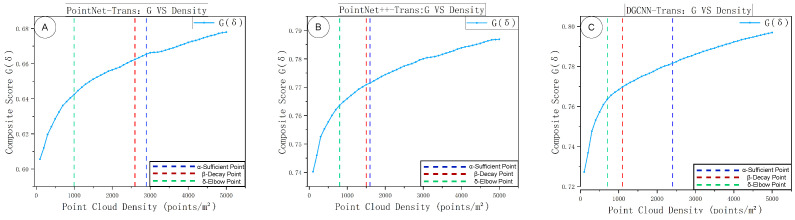
Comprehensive score versus sampling density for three models. (**A**–**C**) PointNet–Trans, PointNet++–Trans, and DGCNN–Trans.

Building on the criterion implementation in Section 3.4.2, this experiment analyzes the composite score and marginal efficiency of the three fused models across different densities. The specific results are shown in Figure 14 and Figure 15, which respectively depict the composite-score *G*–δ curves and the marginal-efficiency η–δ curves under varying sampling densities. Both sets of curves were obtained under a unified evaluation protocol, with density as the only changing variable.

In Figure 15, panels A–C correspond to the composite-score changes of the three models. The three vertical dashed lines mark the threshold types—green for the “knee,” red for the “decay point,” and blue for the “sufficiency point.” This “knee–decay–sufficiency” structure is consistent with observations from density-based grid thinning and adaptive preprocessing [3]. For PointNet–Trans, all three thresholds lie further to the right; there remains a relatively long “improvable window” after the knee, η drops to a low regime later, and accuracy sufficiency is reached later as well, indicating higher sensitivity to density. For PointNet++–Trans, the thresholds are leftmost and most tightly spaced: the knee is crossed at low density, and efficiency decay and accuracy sufficiency are reached quickly, reflecting fast saturation and efficiency priority. DGCNN–Trans falls in between: its knee precedes that of PointNet–Trans but lags behind PointNet++–Trans; $\delta_{\beta }$ lies in the medium-to-high density range and $\delta_{\alpha }$ is moderate, implying continued but slowing gains beyond medium density. Across all three panels, the ordering “green left, red center, blue right” holds, while the spacing between thresholds reveals the relative density demand: PointNet–Trans > DGCNN–Trans > PointNet++–Trans. The corresponding values are reported in Table 6.

**Figure 16:**
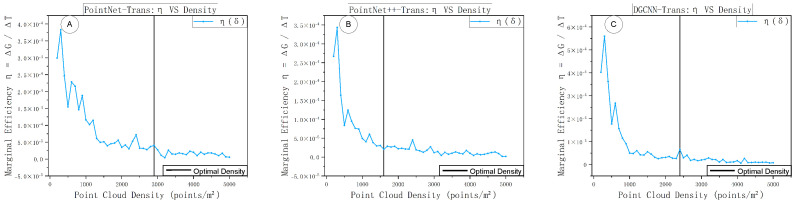
Marginal efficiency versus sampling density for three models. (**A**–**C**) PointNet–Trans, PointNet++–Trans, and DGCNN–Trans.

Panels A–C of Figure 16 likewise depict the marginal-efficiency variations for the three models. In each subplot, the vertical black solid line marks the location of the optimal sampling density. All three plots show that η peaks in the low-density regime, then drops rapidly and oscillates at a near-floor level beyond medium density. Specifically, PointNet++–Trans has the leftmost peak with the fastest decay, and $\delta_{\mathrm{opt}}$ lies at a medium density; DGCNN–Trans decays more gradually, with $\delta_{\mathrm{opt}}$ in the medium-to-high range; PointNet–Trans still yields usable gains before higher densities, so its $\delta_{\mathrm{opt}}$ is the rightmost. This distribution is consistent with the relative ordering of thresholds in Figure 15, indicating that the optimal density is jointly determined by the “efficiency turning point + accuracy sufficiency.”

Aggregating the data from all three models yields recommended densities of approximately $2.8–3.0\times 10^{3}$, $1.5–1.7\times 10^{3}$, and $2.3–2.5\times 10^{3}$ points/m^2^ for the three models, respectively. A cross-model recommended range can thus be set to 1600–2900 points/m^2^, with a robust point estimate of $\delta^{*}\approx 2.3–2.4\times 10^{3}$ points/m^2^. Because density is the sole independent variable under a unified protocol, the band above should be read as an engineering prior calibrated for indoor interiors (matte finishes, typical occlusion, high-SNR handheld). With stronger absorption/specularity, heavier clutter, or lower SNR, the sufficiency and knee thresholds tend to shift right; large homogeneous planar regions may shift left. A short project-level check on one–two representative tiles can locate a scene-specific $\delta^{*}$ without changing architectures or retraining, keeping the rule model-agnostic yet naturally adaptable across materials, occlusion, and sensor noise. This interval avoids the pronounced misclassification associated with low densities while limiting redundant sampling and processing overhead at high densities. Floors dominated by planar components may adopt values near the lower bound, whereas those with dense beams/columns or complex details may move toward the upper bound. Table 7 lists each model’s optimal-density identifier, as well as the cross-model consensus interval and the robust point estimate for direct deployment.

#### 4.3.4. Real-World Scan-to-BIM Deployment

To demonstrate end-to-end practicality under the same unified protocol, we report a concise field deployment on a standard residential floor (area 4511 m^2^) from the same project. Three workflows are compared: (i) a manual baseline (human-led surveying/acceptance), (ii) an unprocessed high-density pipeline (13,034 pts/m^2^), and (iii) a selector-guided pipeline operating at 2438 pts/m^2^ within the recommended band. Table 8 summarizes scan/acceptance time, per-backbone inference time (PointNet–Trans/PointNet++–Trans/DGCNN–Trans), accuracy (OA/mIoU), total points, and throughput. The selector-guided setting preserves accuracy while substantially reducing capture and compute time, yielding markedly higher throughput for Scan-to-BIM without changing architectures or the measurement stack.

All times are wall-clock under the same protocol and hardware. Throughput is reported in m^2^/min and computed as 4511/((scan+inference)/60). Points are density × area (4511 m^2^). Inference times are reported per backbone; OA/mIoU is evaluated on the same area with manually verified labels. For the manual baseline, “Acceptance time” denotes field acquisition/QA by human operators (no inference).

The selector-guided workflow yields substantial end-to-end efficiency gains while keeping accuracy essentially unchanged. Throughput rises from 57.4 to 298.9 m^2^/min (≈5.21×) relative to the unprocessed workflow, and is ≈2.70× higher than the manual baseline (110.6 m^2^/min). These gains come from both capture and compute: scan time drops from 24.3 to 5.1 min (≈4.8× faster), PointNet++–Trans inference shortens from 54.3 to 9.99 min (≈5.4×), and total points reduce from 58.8 M to 11.0 M (≈81% fewer). Accuracy remains within a tight band: for PointNet++–Trans and DGCNN–Trans the deltas are only −0.003/−0.003 and −0.001/−0.001 (OA/mIoU), respectively, while PointNet–Trans improves by +0.068/+0.147, consistent with fewer oversampled neighborhoods benefiting its aggregation. The manual baseline attains high OA (0.92) but lower mIoU (0.72) because boundary ambiguity, occlusions, and class imbalance penalize IoU more strongly than overall accuracy during fast on-site QA; to avoid incidental effects, the present study reports the manual metrics as averages over multiple repeated runs by the same team on the same area.

## 5. Discussions

### 5.1. Method Effectiveness and Value

Unlike prior efforts that improve backbones under a fixed input or thin points by heuristics without accounting for runtime, memory, and power, we elevate sampling density to a first-class acquisition parameter under a unified protocol and quantify its effect jointly on segmentation quality and system cost. We deliberately keep backbones fixed so that density—not model changes—is the causal factor; this yields auditable guidance for acquisition planning and compute budgeting and remains valid even when the backend model is upgraded. The proposed three-criterion selector—accuracy sufficiency, efficiency decay, and the knee point—turns scan-resolution choice from a rule-of-thumb into a computable and auditable decision independent of the specific network and training recipe. Class- and geometry-aware analyses explain why planar elements saturate at moderate density while slender details benefit from denser neighborhoods, which motivates spatially non-uniform allocation rather than uniform oversampling. Framed for a Sensors audience, density becomes a controllable knob on the sensing side that is co-tuned with throughput, memory footprint, and battery endurance, so the same planning rules apply even when the backend model is upgraded. In practice, our recommendations translate directly to Scan-to-BIM workflows and field deployment: teams can plan station counts and dwell times with predictable accuracy targets, document settings for repeatability across floors and projects, and reconcile algorithmic gains with operator workload and device constraints. We provides a field case showing that the same rule yields tangible savings in scanning and compute while preserving accuracy, thereby clarifying how to apply the selector in Scan-to-BIM practice.

Mechanistically, two forces shape the response curve. A geometric visibility threshold determines whether neighborhoods are complete enough for stable normals, curvatures, and boundaries; at low density, boundaries fragment and confusion rises near thin walls, sharp corners, and beam–wall junctions, while moderate densification restores local continuity and reveals fine details. In parallel, time and memory scale roughly linearly with added points; after a task-specific turning region, the discriminative content per point diminishes, marginal efficiency drops, and the curve bends at the knee. We discretize this continuum into actionable thresholds: the sufficiency point caps “how much is enough” for the task, the decay point guards deployability under latency and power budgets, and the knee offers a robust default when decisions must be made quickly. Because density needs are class- and region-specific, the preferred strategy is to steer density by geometric salience and model uncertainty—reinforcing junctions, thin walls, and persistent occlusions while relaxing large homogeneous slabs and ceilings—and to co-optimize with tile size, overlap, batching, and scheduling so peak memory remains within device limits. Treating density as a documented parameter within a sensing–computing pipeline, rather than a hidden constant, improves reproducibility, clarifies accountability in accuracy–efficiency trade-offs, and strengthens the linkage between sensing operations and downstream BIM verification.

### 5.2. Limitations and Future Work

Our findings, though robust within the present scope, are bounded by one project, one primary device, and indoor scenes with controlled motion. We respectfully acknowledge that the present validation does not yet span diverse building typologies, sensors, and environments (e.g., outdoor facades, cluttered interiors), due to practical constraints on data collection and compute; we have planned follow-on studies across building types and sensors and will report how the three thresholds shift, with confidence bounds, under varied conditions. Broader validation is required across structural systems, construction stages, finishing materials, and sensor models to confirm the generality of the 1600–2900 points/m^2^ operating band and to map how noise characteristics shift $\left(\delta_{\alpha },\delta_{\beta },\delta_{\mathrm{knee}}\right)$. Mechanistically, low-reflectance or glossy surfaces and heavy clutter typically increase the density needed for stable neighborhoods (raising the sufficiency point), whereas large homogeneous planes and high-SNR devices may lower it. We therefore recommend reporting the decision rule together with the per-project $\delta^{*}$ obtained via the same selector in Section 3.4. This lightweight recalibration keeps density as the only manipulated variable and yields auditable, transferable guidance without introducing new backbones or theoretical models. Challenging conditions—outdoor facades, reflective or dark surfaces, dynamic crews, strong occlusions—may alter sufficiency and decay thresholds in ways not fully captured here; establishing confidence intervals for the recommended density will improve portability across jobsites. On the learning side, device/domain adaptation together with semi- and self-supervised strategies can reduce annotation burden and quantify device-induced shifts in the selector. We also did not benchmark very recent segmentation backbones (e.g., MinkowskiNet, RandLA-Net, Point Transformer) in this revision. This is a deliberate design choice to isolate the causal effect of sampling density under one training/measurement pipeline; adding models with different sparsity kernels or sampling policies would entangle density with architecture capacity and framework-level compute optimizations. In future work, we will port these backbones to our unified protocol and report how sufficiency and knee thresholds shift when the backend is upgraded, thereby complementing the present, architecture-agnostic guidance. We will also examine criterion stability via sensitivity of the composite score *G* to its weights, agreement among alternative metrics, and threshold variance across datasets, yielding parameter-setting guidance and practical bounds for $\delta^{*}$.

For deployment at the system level, the selector should be fused with scan planning to form a stationing–scanning–inference loop where density is schedulable and co-optimized with memory, latency, and energy on edge hardware. We will explore incremental and streaming inference, adaptive batching and tile prefetching to meet on-site deadlines, and joint optimization of survey routes and local density to minimize walking distance and idle time. Beyond semantic segmentation, we plan to extend the framework to instance parsing, component topology recovery, and geometric reconstruction tied to BIM acceptance, evaluating impact with a triad of semantic accuracy, geometric deviation, and processing time. Expected deliverables include cross-project consistency reports and open tools, reference presets for complex zones, and lightweight plugins that recommend scanner settings or thinning levels in real time given device and schedule constraints. Collectively, these steps aim to consolidate the density–efficiency trade-off into deployable rules that raise reliability and throughput in real construction practice.

## 6. Conclusions

This study introduces a unified evaluation pipeline that decouples sampling density from architectural and training factors and frames indoor LiDAR segmentation within a density–accuracy–efficiency triad. Using three computable criteria—accuracy sufficiency, efficiency decay, and the knee point—together with a marginal-gain measure, density choice becomes programmable rather than heuristic. Empirically, composite performance rises rapidly and then saturates as density increases, while marginal efficiency falls sharply beyond a mid-density regime. Errors concentrate around beam–wall interfaces, occluded regions, and thin members; planar classes stabilize at moderate densities, whereas beam-like elements remain more density-sensitive. Aggregating three backbones yields an operating band of approximately 1600–2900 points/m^2^, with a robust nominal setting near 2400 points/m^2^, balancing accuracy with time, memory, and power. For deployment, planar-dominant floors can adopt the lower bound to curb latency, while detail-rich or risk-prone zones should approach the upper bound; under a fixed point budget, prioritize structure-sensitive areas via targeted local densification rather than uniform oversampling. Overall, the framework converts scan-resolution selection into a quantitative and auditable decision, offering direct guidance for scan planning, compute allocation, and on-site quality assurance.

## Figures and Tables

**Table 1:** Training and evaluation protocol summary.

Item	Setting
Batch size	32
Points per sample (*N*)	8192
Epochs	500
Optimizer	Adam (lr $1\times 10^{-3}$, weight decay $5\times 10^{-4}$)
LR Scheduler	OneCycleLR
Early stopping	patience =10
EMA	Enabled
Data augmentation	Rotation (Z), scaling, translation, jitter, point drop
Loss	Weighted cross-entropy
Metrics (val.)	OA, mIoU, macro-F1
Mixed precision	AMP on

**Table 2:** Dataset and split statistics.

Category	Item	Setting
Train/Val	Source	Synthetic (BIM) tiles, Building #10
	Floors	B1, 10–30
	Tiles (approx.)	>50,000 (total)
	Labels	BIM-derived per-point
	Classes	4 (Wall/Column, Beam, Floor, Ceiling)
Test	Source	Real site scans (multi-station ICP)
	Floors	5–9 (standard)
	Tiles (approx.)	— (tiled at inference)
	Labels	Manually verified
	Classes	Same as above

**Table 3:** Per-class performance of PointNet–Trans.

Class	IoU	Precision
Wall and Columns	0.777	0.852
Beam	0.409	0.474
Floor	0.393	0.989
Ceiling	0.680	0.812
Average IoU	0.565	—
Overall Accuracy	—	0.771

**Table 4:** Per-class performance of PointNet++–Trans.

Class	IoU	Precision
Wall and Columns	0.779	0.864
Beam	0.406	0.502
Floor	0.903	0.975
Ceiling	0.841	0.985
Average IoU	0.732	—
Overall Accuracy	—	0.856

**Table 5:** Per-class performance of DGCNN–Trans.

Class	IoU	Precision
Wall and Columns	0.778	0.815
Beam	0.420	0.559
Floor	0.875	0.983
Ceiling	0.819	0.991
Average IoU	0.723	—
Overall Accuracy	—	0.854

**Table 6:** Per-model thresholds for density selection.

Model	$\delta_{\alpha }$ (Sufficiency)	$\delta_{\beta }$ (Decay)	$\delta_{\mathrm{knee}}$ (Knee)
PointNet–Trans	2900	2600	1000
PointNet++–Trans	1600	1500	800
DGCNN–Trans	2400	1100	700

**Table 7:** Consensus recommendations for sampling density across models.

Category	Item	Value (Points/m^2^)
Per-model	PointNet–Trans	2900
	PointNet++–Trans	1600
	DGCNN–Trans	2400
Cross-model	Consensus band	[1600,2900]
	Recommended density	2400

**Table 8:** End-to-end Scan-to-BIM deployment summary.

Workflow	Measure	Value
Manual baseline	Acceptance time	40.8 (min)
	OA/mIoU	0.92/0.72
	Throughput	110.6 (m^2^/min)
Unprocessed	Density	13,034 (pts/m^2^)
	Scan time	24.3 (min)
	Inference—PointNet–Trans	52.8 (min)
	Inference—PointNet++–Trans	54.3 (min)
	Inference—DGCNN–Trans	56.1 (min)
	OA/mIoU—PointNet–Trans	0.771/0.565
	OA/mIoU—PointNet++–Trans	0.856/0.732
	OA/mIoU—DGCNN–Trans	0.854/0.723
	Points	58.8 M
	Throughput	57.4 (m^2^/min)
Selector-guided	Density	2438 (pts/m^2^)
	Scan time	5.1 (min)
	Inference—PointNet–Trans	9.72 (min)
	Inference—PointNet++–Trans	9.99 (min)
	Inference—DGCNN–Trans	10.33 (min)
	OA/mIoU—PointNet–Trans	0.839/0.712
	OA/mIoU—PointNet++–Trans	0.853/0.729
	OA/mIoU—DGCNN–Trans	0.853/0.722
	Points	11.0 M
	Throughput	298.9 (m^2^/min)

## Data Availability

The data supporting the findings of this study are not publicly available due to project confidentiality. Portions of the datasets are included within the article, and some core algorithms are presented in the form of pseudocode in the manuscript. Additional details may be obtained from the corresponding author upon reasonable request.

## Abbreviations

The following abbreviations are used in this manuscript:

LiDAR	Light Detection and Ranging
DGCNN	Dynamic Graph Convolutional Neural Network
MLP	Multi-Layer Perceptron
CE	Cross-Entropy
